# Suppression of IGF-I signals in neural stem cells enhances neurogenesis and olfactory function during aging

**DOI:** 10.1111/acel.12365

**Published:** 2015-07-29

**Authors:** Zayna Chaker, Saba Aïd, Hugues Berry, Martin Holzenberger

**Affiliations:** 1 INSERM, Centre de Recherche UMR938, Hôpital Saint-AntoineParis, 75012, France; 2 Sorbonne Universités, UPMC – Université Pierre et Marie CurieParis, 75005, France; 3 Faculté de Médecine, Université Paris DescartesParis, 75006, France; 4 INRIA and CNRS UMR 5205, Université de LyonVilleurbanne, 69621, France

**Keywords:** aging, Cre-loxP system, energy metabolism, IGF-I, neurogenesis, olfaction

## Abstract

Downregulation of insulin-like growth factor (IGF) pathways prolongs lifespan in various species, including mammals. Still, the cellular mechanisms by which IGF signaling controls the aging trajectory of individual organs are largely unknown. Here, we asked whether suppression of IGF-I receptor (IGF-1R) in adult stem cells preserves long-term cell replacement, and whether this may prevent age-related functional decline in a regenerating tissue. Using neurogenesis as a paradigm, we showed that conditional knockout of IGF-1R specifically in adult neural stem cells (NSC) maintained youthful characteristics of olfactory bulb neurogenesis within an aging brain. We found that blocking IGF-I signaling in neural precursors increased cumulative neuroblast production and enhanced neuronal integration into the olfactory bulb. This in turn resulted in neuro-anatomical changes that improved olfactory function. Interestingly, mutants also displayed long-term alterations in energy metabolism, possibly related to IGF-1R deletion in NSCs throughout lifespan. We explored Akt and ERK signaling cascades and revealed differential regulation downstream of IGF-1R, with Akt phosphorylation preferentially decreased in IGF-1R^−/−^ NSCs within the niche, and ERK pathway downregulated in differentiated neurons of the OB. These challenging experimental results were sustained by data from mathematical modeling, predicting that diminished stimulation of growth is indeed optimal for tissue aging. Thus, inhibiting growth and longevity gene IGF-1R in adult NSCs induced a gain-of-function phenotype during aging, marked by optimized management of cell renewal, and enhanced olfactory sensory function.

## Introduction

In higher organisms, adult tissues harbor stem cells that generate lifelong new differentiated cells, ensuring long-term repair and individual longevity. This process has been extensively studied in the adult mouse brain, since the discovery of neural stem and progenitor cells (neural stem cell – NSC) (Doetsch *et al*., 1999; Doetsch, 2003). It is well established that adult neurogenesis contributes substantially to structural and functional plasticity of the brain during physiological aging. However, the extrinsic signals modulating neuronal replacement throughout lifespan are still largely unknown. Recent studies suggest that circulating factors constitute a systemic aging milieu capable of regulating and coordinating tissue regeneration. Indeed, some long-lived mouse strains such as Ames dwarf or Laron mutants, with typically low systemic levels of growth hormone and insulin-like growth factor I (IGF-I), show increased neurogenesis and preserved hematopoietic stem cell (HSC) pool (Sun *et al*., 2005; Ratajczak *et al*., 2011). Thus, genes known to regulate growth and aging seem to also control regenerating cell number over time (Sharpless & DePinho, 2007). Because of its ubiquitous action and the remarkable plasticity of neuroendocrine regulation, IGF-I acts as pivotal effector that translates fluctuating environmental conditions into endogenous molecular signals in a variety of cell types (Fernandez & Torres-Alemán, 2012). Importantly, IGF-I in the brain modulates lifespan in mammals (Holzenberger *et al*., 2003; Kappeler *et al*., 2008) and is also a powerful cell proliferation, differentiation, and survival factor (Aberg *et al*., 2000; Kalluri *et al*., 2007). IGF-I receptor (IGF-1R) is expressed in adult NSCs and strongly enriched in neuronal precursors migrating toward the olfactory bulb (OB) (Arsenijevic & Weiss, 2001; Ziegler *et al*., 2014). In this study, we investigated short- and long-term effects of IGF-I signaling on adult NSCs, showing how this important energy-sensing pathway regulates lifelong management of neuronal replacement. Using a novel transgenic mouse model, we inactivated IGF-I signaling specifically in adult NSCs, and traced knockout cell lineages from the subventricular zone (SVZ) to OB by monitoring a fluorescent reporter. This mouse model faithfully recorded the cumulative effects of IGF-1R deletion from adult NSCs on cell replacement. We analyzed long-term consequences of the receptor mutation at the level of single cells, tissue architecture, organ function, and physiology. This study concludes that lifelong exposure of NSCs to IGF-I accelerates the age-related decline of adult neurogenesis in the olfactory system, entailing noticeable changes in sensory function and possibly also in metabolic regulation.

## Results

### Knockout of IGF-1R in adult NSCs delays age-related decline of OB neurogenesis

**Fig 1:**
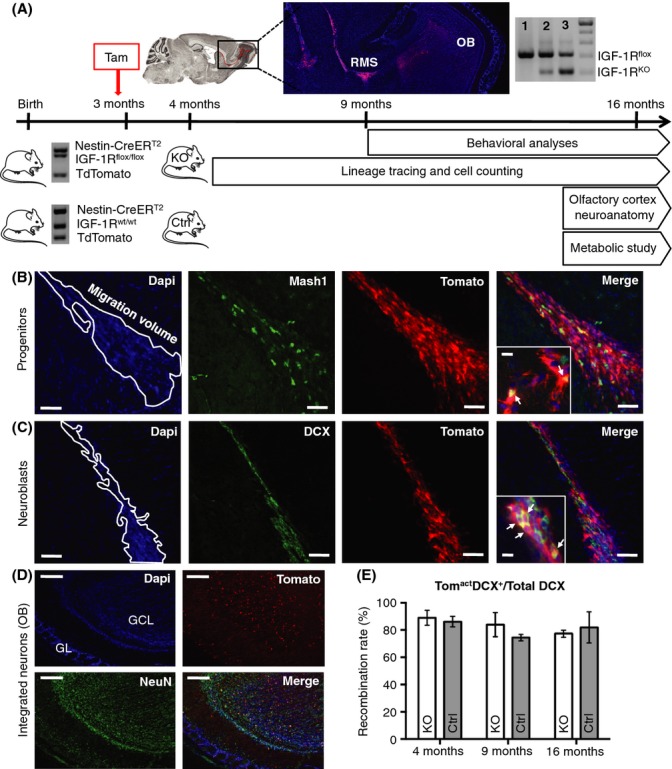
Lineage tracing of adult-born IGF-1R knockout cells in the RMS. (A) Experimental design. Mice carried Nes-CreER^T^^2^ and tdTomato transgenes, combined with conditional IGF-1R^flox/flox^ alleles in the mutant group and IGF-1R wild-type alleles in controls. Tamoxifen (Tam) was i.p. injected for KO induction at 3 months. Lineage tracing allowed quantitative analysis of regenerating cells at 4, 9, and 16 months. Behavioral studies were performed at 9 and 16 months and analysis of olfactory bulb (OB) neuroanatomy and metabolic study at 16 months. Cre-lox recombination rate was determined by PCR on 16-month-old OB and was 0% for a noninduced IGF-1R^flox/flox^ control (lane 1) and 15.7 ± 0.3% for mutant OB (lane 2). A sample from heterozygous IGF-1R^flox/^^KO^ animal (50% of alleles recombined) was used to calibrate the assay (lane 3). (B and C) Representative sagittal sections of the migratory stream (outlined), with magnifications showing triple-labeled cells (arrows). Scale bars, 50 and 10 μm, respectively. Newborn Tom^act^ neural progenitors were labeled with anti-Mash1 antibody (B) and newborn Tom^act^ neuroblasts with anti-DCX antibody (C). (D) Representative sagittal sections of olfactory bulb at 4 months. Adult-born Tom^act^NeuN^+^ neurons integrate into granular (GCL), mitral (MCL), and glomerular (GL) cell layers. Scale bars, 400 μm. (E) Recombination rate over time was determined dividing Tom^act^DCX^+^ colocalized volume by DCX^+^ volume in the RMS (*n* = 6 at 4, 9, and 16 months).

To specifically target adult NSCs, we used a nestin-CreER^T2^ transgene (Lagace *et al*., 2007) expressing tamoxifen (Tam)-inducible Cre recombinase selectively in these cells and crossed it into mice that carry a floxed IGF-1R gene and a Cre-inducible tdTomato knock-in cell lineage marker. These nestin-CreER^T2^;CAG-tdTomato^+/0^;IGF-1R^flox/flox^ mice and their controls (nestin-CreER^T2^;CAG-tdTomato^+/0^;IGF-1R^wt/wt^) were injected with Tam at 3 months of age, inducing loss of IGF-1R and intense red label in all NSCs and their cell progeny born after Cre induction. We demonstrated complete absence of ectopic Cre-lox recombination from peripheral tissues of tamoxifen-treated mutants (Fig. S1A,B, Supporting information). Given its high specificity, nestin-CreER^T2^-driven conditional mutagenesis allowed studying the role of IGF-1R in adult-born neurons by comparing labeled cell lineages between mutant and control brains (Fig.1A). Of note, recombination indicative of neurogenesis in dentate gyrus and hypothalamus was orders of magnitude lower than that in SVZ in this mouse model (Fig. S1C). In the olfactory regenerative system, we followed progenitors and neuroblasts throughout the rostral migratory stream (RMS) using cell-type-specific markers in addition to tdTomato fluorescence (Fig.1B,C). We also studied newborn mature neurons (NeuN^+^Tom^act^) that had integrated into the olfactory bulb (Fig.1D). At 4 months of age, over 90% of migrating neuroblasts showed Tam-induced Cre-lox recombination in mutants and controls (Fig.1E). Although slightly decreasing with age, this recombination rate remained similar in both groups, indicating that the same proportion of recombined cells was traced in all animals throughout this longitudinal study. At 16 months, we detected a prevalence of 15.7% ±0.3 genomic recombination in mutant OB (Fig.1A), concordant with the proportion of cells having been replaced at that age, which is about 0.85 ± 0.06 × 10^6^ Tom^act^ cells over 6.44 ± 0.44 × 10^6^ total (Parrish-Aungst *et al*., 2007). To validate the hypothesis that IGF-I signaling impacts on age-related depletion of regenerating cells, we performed histological analyses at 1, 6, and 13 months after Tam administration.

**Fig 2:**
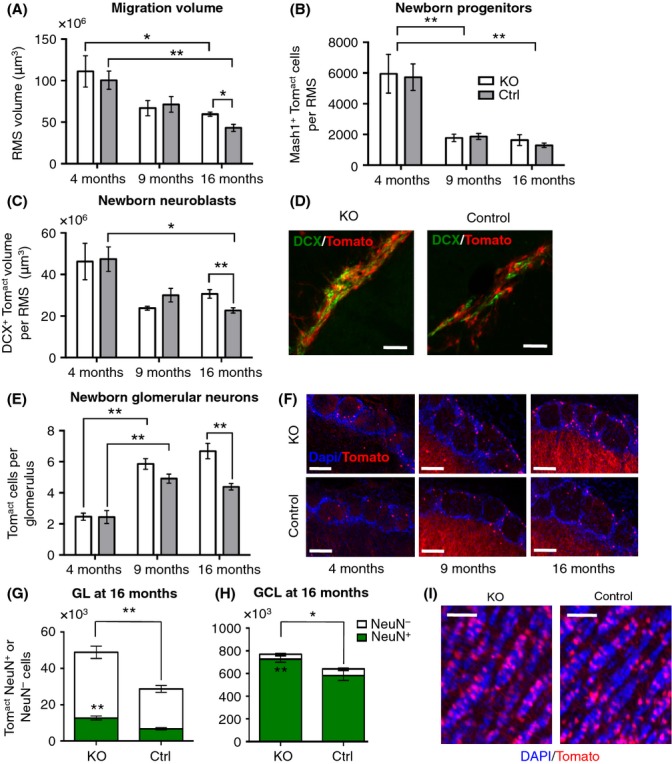
Inhibition of IGF-I signaling in adult NSCs prevents age-related decrease in neurogenic potential. (A) RMS volume was measured on DAPI-counterstained sections. At 16 months, *P*_*KO-Ctrl*_ = 0.015, Mann–Whitney *U-*test, *n* = 6. (B and C) Number of regenerating cells in the RMS. (C) Newborn neuroblasts at 16 months. *P*_*KO-Ctrl*_ = 0.002, Mann–Whitney *U*-test, *n* = 6. (D) Representative sagittal sections of Tom^act^DCX^+^ neuroblasts in the RMS at 16 months. Scale bars, 20 μm. (E) Evolution with age of the average number of newborn cells integrated into each glomerulus. At 16 months, *P*_*KO-Ctrl*_ = 0.004, Mann–Whitney *U*-test, *n* = 6. (F) Representative sagittal sections of KO and control glomeruli at 4, 9, and 16 months. Scale bars, 100 μm. (G) NeuN^+^ cells represent 25% of total Tom^act^ cells in mutant GL and 21% in control. Number of Tom^act^ and of Tom^act^NeuN^+^ cells were significantly increased in mutants compared with controls. *P *=* *0.004, Mann–Whitney *U*-test, *n* = 6. (H) Proportion of NeuN^+^ newborn cells was more important in GCL than in GL (91–94% for both genotypes). Number of Tom^act^ and of NeuN^+^Tom^act^ cells were significantly increased in mutants compared with controls. *P*_*Tom*_^*act*^ = 0.034 and *P*_*Tom*_^*act*^_*NeuN*_^*+*^ = 0.007, Mann–Whitney *U*-test, *n* = 7. (I) Representative micrographs of DAPI-counterstained coronal GCL sections with Tom^act^ newly integrated cells. Scale bars, 50 μm.

Neural progenitors and neuroblasts migrate from adult SVZ to OB, forming the RMS. Delimited by glial tubes, RMS volume is tightly controlled and decreases strongly with age (Mobley *et al*., 2013). Most studies on rodent neurogenesis use males, and hence, we here present this group first. As expected, RMS volume decreased significantly between 4 and 16 months of age, in mutants and controls (Fig.2A). Both groups had similar volume at 4 months, but age-related decline was slower in mutants. From 4 to 16 months, volume decreased 57% in controls vs. 46% in KO, resulting in 38% larger RMS volume in mutants at 16 months (59 ± 2 × 10^6^ vs. 43 ± 4 × 10^6^ μm^3^, *P *=* *0.015; Fig.2A). We then asked whether the suppression of IGF-I signaling in adult NSCs modifies progenitor or neuroblast proportions in the RMS. In accordance with declining RMS volumes, we observed a significant age-related decrease of progenitor and neuroblast populations in mutants and controls (Fig.2B,C). In both groups, the number of progenitors declined dramatically between 4 and 9 months (mutant: 5728 ± 862 vs. 1780 ± 238 and control: 5926 ± 1258 vs. 1872 ± 194 cells; Fig.2B) and remained stable thereafter until 16 months. Thus, long-term maintenance of progenitor cell compartment did not depend on IGF signaling. Concerning neuroblasts, mutants and controls displayed the same volume of migrating cells at 4 months. However, while controls showed significant depletion between 4 and 16 months (−52%), the long-term loss was limited to −34% in mutants (Fig.2C). In consequence, KO brains at 16 months presented with 35% larger volume of migrating neuroblasts (30 ± 2 × 10^6^ vs. 22 ± 1 × 10^6^ μm^3^, *P *<* *0.01; Fig.2C,D). Thus, the deletion of IGF-1R in adult NSCs had clearly distinguishable effects on progenitor vs. neuroblast compartments, efficiently protecting the latter from age-related decline.

Not all immature neurons reaching the OB do integrate granular (GCL) or glomerular (GL) layers as new neurons. We therefore checked whether OB neuronal compartments were affected by IGF-1R deletion in upstream adult NSCs. Between 4 and 9 months, the number of newborn glomerular neurons increased substantially in KO and control animals (Fig.2E,F). Importantly, with increasing age, the number of new neurons progressively differed between groups (Fig.2E). While in controls the number of newly generated neurons integrating glomeruli plateaued at 9 months (9 months: 4.9 ± 0.3 vs. 16 months: 4.4 ± 0.2 cells per glomerulus), it continued to increase in mutants between 9 and 16 months (5.8 ± 0.3 vs. 6.7 ± 0.5 cells per glomerulus). As a result, 16-month-old mutants exhibited significantly more new cells per glomerulus (Fig.2E) and also contained conspicuously more newborn glomerular cells in the entire GL (+70%, *P *=* *0.004, Fig.2G). In the adult brain, not all mature glomerular cells are expressing neuronal marker NeuN (Winpenny *et al*., 2011). In accord with that, we found a similar proportion of total NeuN^+^ newborn glomerular neurons in mutants and controls (25% ±2 and 21% ±2, respectively; Fig.2G). As expected, the proportion of NeuN^+^ neurons was much higher in GCL, with no significant difference between KO and controls (91% ±1 in controls and 94% ±1 in mutants; Fig.2H). Similar to GL, GCL at 16 months contained 20% more adult-born cells in mutants compared with controls (*P *<* *0.01; Fig.2H,I). These data indicated that durable suppression of IGF signaling in adult NSCs supported lifelong neurogenesis by selectively fostering adult-born populations of neuroblasts and neurons. Interestingly, Tom^act^Mash1^+^ cell number was not affected by IGF-1R deletion (Fig.2B), suggesting that increased neurogenesis in mutants occurred without depletion of progenitors.

We performed the same analyses in females where turnover of neuronal progenitors and replacement of other differentiated cell types are known to be higher compared with males (Shingo *et al*., 2003; Leeman & Brunet, 2014; Nakada *et al*., 2014). IGF-1R deletion from adult NSCs in females did not affect age-related changes in RMS volume (Fig. S2A). Moreover, and in contrast to female controls and to all males, IGF-1R knockout females at 4 months displayed significantly less migrating progenitors (−48%, *P *<* *0.05) and a smaller volume of migrating neuroblasts (−28%, *P *<* *0.01; Fig. S2B,C). Cleaved caspase-3 staining ruled out that this was due to increased cell death in SVZ of female mutants (Fig. S2D,E). Thus, reduced number of neural progenitors in 4-month-old female KO forebrain resulted from diminished cell proliferation, as confirmed by Ki67-Mash1 double staining in RMS (Fig. S2F,G). Regarding age-related changes in the number of progenitor cells (Fig. S2B), this population was progressively depleted from 4 to 16 months in control females (4 months: 6066 ± 658; 9 months: 2504 ± 632; 16 months: 1014 ± 152 progenitor cells, *P *<* *0.05). In stark contrast, mutant females maintained young adult numbers of progenitors in the transition from 4 to 16 months (4 months: 3156 ± 606; 16 months: 2654 ± 208). Similarly, only control females experienced significant age-related decline of migrating neuroblast volume between 4 and 9 months (52 ± 4 × 10^6^ vs. 27 ± 5 × 10^6^ μm^3^, *P *<* *0.01; Fig. S2C), whereas mutants did not. Despite reduced number of regenerating cells at 4 months, female mutants tended to accumulate more newborn neurons in GL at 16 months (6.6 ± 0.5 cells per glomerulus in KO vs. 5.6 ± 0.5 in controls; Fig. S2H).

### IGF-1R preferentially activates Akt in NSCs and ERK in differentiated neurons

Increased number of newborn neurons in the OB of mutants can be due to enhanced neuronal integration, or increased cell production, or both. To investigate these two hypotheses, we focused on MAPK/ERK and Akt/mTOR pathways, controlling cell survival, proliferation, and differentiation, downstream of IGF-1R. In males, where differences in cell number were most significant, we measured Akt and ERK activation in differentiated neurons at 16 months, and in NSCs within the niche at 4 months, when proliferative activity is the highest. P-ERK was significantly reduced in western immunoblots of mutant OB, while P-Akt remained unchanged (Fig. S3A,B). Using immunohistochemistry, we found the number of Tom^act^P-ERK^+^ cells decreased in mutants (−51%; *P *=* *0.03; Fig. S3C,D), while other P-ERK^+^ cell types (i.e. Tom^−^) were as abundant as in controls (Fig. S3D). This finding confirmed that reduced P-ERK was selectively due to IGF-1R inactivation in KO newborn neurons and thus most likely a cell-autonomous effect. Several studies suggest that ERK activation mediates neuronal damage in pathological contexts (Subramaniam *et al*., 2004) and that ERK inhibition can be beneficial for normal neuronal survival and function (Ounallah-Saad *et al*., 2014). Decreased ERK activation as we observed in mutants might ensure neuronal resistance and structural plasticity, thereby facilitating cell integration. In contrast to OB at 16 months, mutant SVZ at 4 months contained very few Tom^act^P-ERK^+^ cells, with no significant difference between mutant and control (KO: 133 ± 28 vs. controls: 172 ± 45 cells mm^−2^). Importantly, at this same age, number of Tom^act^P-Akt^+^ NSCs was substantially reduced in mutants (−66%; *P *=* *0.028; Fig. S3E,F). This suggested that in adult NSCs, ERK signaling was much less affected by IGF-1R deletion than Akt, in agreement with a study on GH pathway inhibition in hippocampal neural precursors (Devesa *et al*., 2014). We next wanted to know whether less P-Akt in IGF-1R^−/−^ NSCs had an effect on long-term stem cell maintenance. In mutants, the pool of GFAP^+^Tom^act^ cells increased between 4 and 16 months, while controls exhibited the expected age-related decrease in NSC number (Fig. S3G). This reciprocal dynamics resulted in twice as many stem cells in mutant SVZ at 16 months compared with controls (KO: 1240 ± 130 vs. controls: 570 ± 130 cells mm^−2^; *P *=* *0.032; Fig. S3G). Combined signaling data obtained in OB and SVZ indicated that the deletion of IGF-1R has differential effects on neurons and NSCs.

Taken together, male and female data demonstrated that the inhibition of IGF-I signaling in adult NSCs at early adulthood protected olfactory neurogenesis from age-related decline, supposedly by regulating stem cell self-renewal vs. differentiation over age, and eventually increasing neuronal survival and integration in the OB. This beneficial effect of reduced growth factor signaling on maintenance of regenerative compartments with age was counterintuitive with respect to the confirmed role of IGF signaling as strong promoter of cell proliferation, survival, and widespread tissue regeneration factor (Anderson *et al*., 2002; Kalluri *et al*., 2007). To clarify this point, we used mathematical modeling and designed a 4-compartment proliferating paradigm predicting optimal growth factor levels over time.

### *In silico* modeling of growth factor-dependent neurogenesis

**Fig 3:**
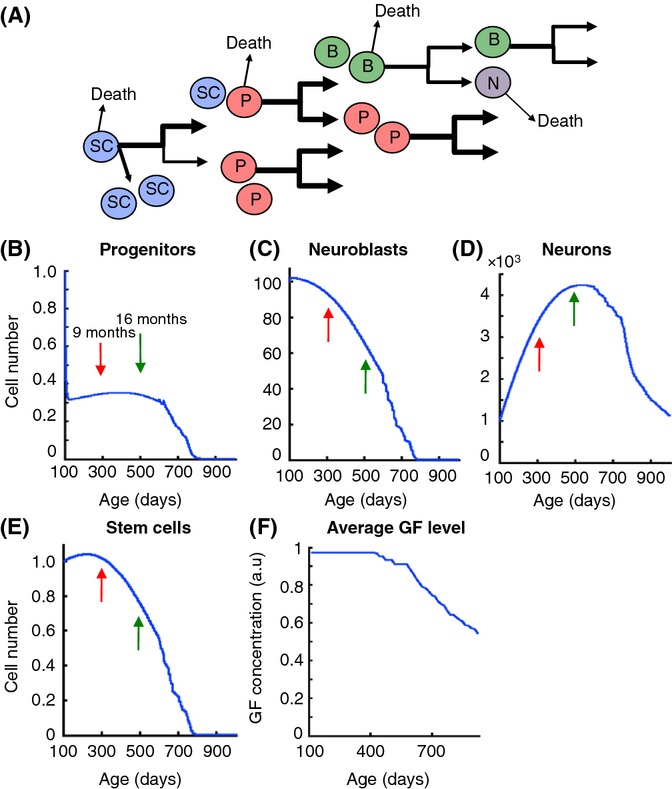
Modeling cell replacement: Downregulation of growth factor stimulation (*GF*) is optimal for tissue maintenance during aging. (A) Schematic representation of the mathematical 4-compartment model. Arrow thickness is proportional to division, differentiation, and death probabilities. (B–D) Age-dependent distributions of progenitor P, neuroblast B, and neuronal N populations. Arrows indicate the time points 9 months (red) and 16 months (green) that were chosen for the experimental study. (E) Stem cell distribution with age as determined from the model. (F) Optimal *GF* level over age assuring maximum regeneration throughout lifespan. *GF* distribution results from an iterative optimization procedure applied every Δ*t* = 10 days and was averaged over 36 Δ*t*.

The model reproduces the characteristics of SVZ neurogenesis in an adult mouse, namely proliferation and differentiation through consecutive compartments representing stem cells (S), progenitors (P), neuroblasts (B), and neurons (N) (Fig.3A). This model was designed so that division, differentiation, and death rates depend on age and growth factor levels (*GF*). For every time step, an optimization procedure selects the *GF* stimulation maximizing a benefit/cost ratio for the aging tissue (details in online methods). The time series obtained *in silico* for P, B, and N populations closely mimics cell population dynamics observed experimentally in controls, regardless of gender. Modeling produced a similar decrease (−60%) in progenitors and a 40% depletion of neuroblasts between 4 and 16 months of age (Fig.3B,C). New neurons accumulate over time, and the model predicted a fourfold increase between 4 and 16 months (Fig.3D). That this exceeds the experimentally determined twofold increase in control males (Fig.2E) and females (Fig. S2H) is likely explained by the fact that our population-based model did not implement specific cell survival and integration rates for newly generated neurons.

*In silico* modeling also predicted that SCs were well preserved between early and mid-adulthood (Fig.3E), in accord with aging studies of adult SVZ (Bouab *et al*., 2011), and their depletion started around day 400. A major output from this theoretical approach was that once an individual is half into adult life, continuously decreasing *GF* level revealed optimal for long-term tissue maintenance (Fig.3F). This *GF* distribution fits with the experimental data obtained in mice and humans, showing a plateau followed by progressive decline of growth hormone and IGF-I levels with advancing age (Bartke *et al*., 2013).

### Increased number of olfactory bulb glomeruli in aged mutants

**Fig 4:**
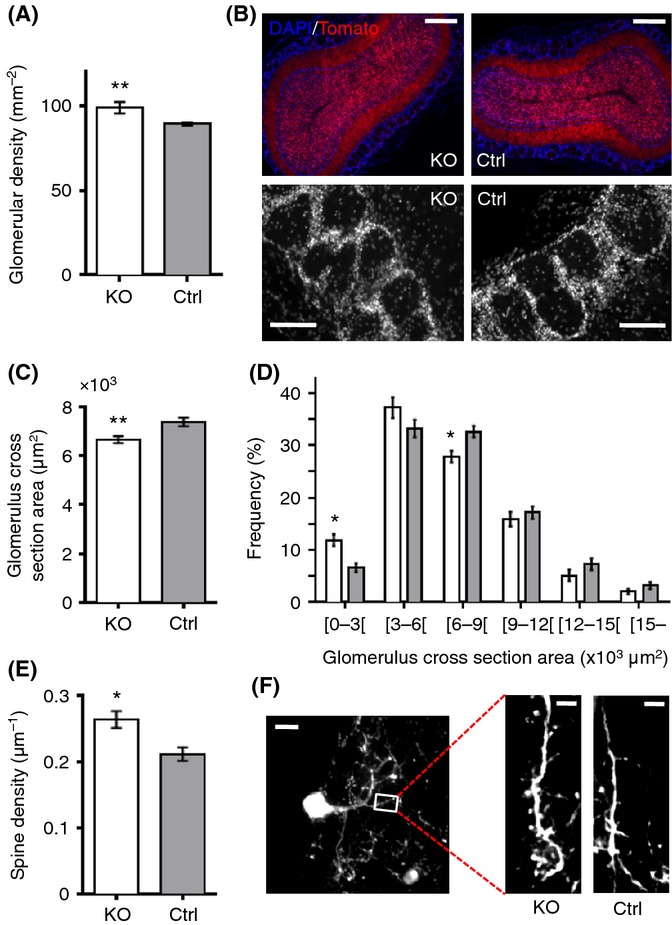
Deletion of IGF-1R in adult-born neurons leads to neuro-anatomical remodeling of olfactory glomeruli. (A) Density of glomeruli in GL. *P *<* *0.001, Mann–Whitney *U*-test, *n* = 7. (B) Coronal sections of olfactory bulb at 16 months (top, scale bars represent 400 μm), with higher magnification of GL (below, scale bars represent 100 μm). (C) Average size of glomeruli. *P *=* *0.009, Mann–Whitney *U*-test, *n* = 7. (D) Size distribution of glomeruli analyzed using repeated-measures ANOVA (RM-ANOVA) and Bonferroni *post hoc* test. *P *<* *0.05 for [0–3000] and [6000–9000] size classes. (E and F) Spine density on secondary dendrites of newborn glomerular interneurons. *P *=* *0.014, Mann–Whitney *U*-test, *n* = 6. (F) Scale bars, 10 (left) and 5 μm (right). All analyses were performed on males.

We showed that ablation of IGF-1R from adult NSCs contributes to maintaining youthful olfactory neurogenesis in an aging brain and asked how aged olfactory bulb structures respond to increased supply with new neurons. While the number of newly born neurons integrating the olfactory bulb had increased, no gross anatomical changes were observed. OB wet weight as well as GL and GCL thickness were the same in mutants and controls (not shown). However, with respect to GL histoanatomical structure, glomerular density was significantly increased in male mutants (98 ± 2 vs. 87 ± 1 glomeruli mm^−2^, *P *<* *0.001; Fig.4A,B), and cross-sectional area of glomeruli was clearly smaller in KO compared with controls (6664 ± 138 vs. 7391 ± 172 μm^2^; *P *<* *0.01; Fig.4C). Accordingly, small glomeruli (< 6000 μm^2^) contributed most to the increased total number of glomeruli in mutants (Fig.4D). We measured glomerular size and density also in females and found no significant difference between KO and controls (cross-sectional area, KO: 6283 ± 346 vs. controls: 6942 ± 240 μm^2^; density, KO: 84 ± 6 vs. controls: 82 ± 3 glomeruli mm^−2^), indicating that penetrance of this glomerular phenotype correlated with the number of IGF-1R-deficient neurons newly integrated in the GL.

To consolidate this unexpected result, we repeated histomorphometric measurements in mice with ubiquitous conditional IGF-1R knockout, induced at 3 months of age (Ubi-IGF-1R^KO/KO^). In 16-month-old Ubi-IGF-1R^KO/KO^ OB, glomerulus surface area was markedly smaller than in Ubi-IGF-1R^wt/wt^ controls (4971 ± 359 vs. 7170 ± 411 μm^2^; Fig. S4A). In those mutants, 30% of glomeruli measured < 3000 μm^2^, compared to just 9% in controls (Fig. S4B). GCL of Ubi-IGF-1R^KO/KO^ mice seemed unaffected, suggesting that remodeling occurred selectively in GL (Fig. S4C). Moreover, morphology of IGF-1R-deficient newborn neurons was marked by increased spine density in both males (2.6 ± 0.1 vs. 2.1 ± 0.1 spines per 10 μm, *P *=* *0.014; Fig.4E,F) and females (2.7 ± 0.1 vs. 2.2 ± 0.1 spines per 10 μm; *P *=* *0.02), pointing to a cell-autonomous effect of IGF-1R deletion. Collectively, these data suggested that adult-born neurons resistant to IGF signaling promote the formation of new functional units in the GL, while such proliferation of glomeruli is typically not seen in adult mice (Pomeroy *et al*., 1990). Thus, lifelong regulation of neurogenesis by IGF is able to trigger developmental processes enhancing GL plasticity in the aging brain.

### Improved olfactory function in aged mutants

**Fig 5:**
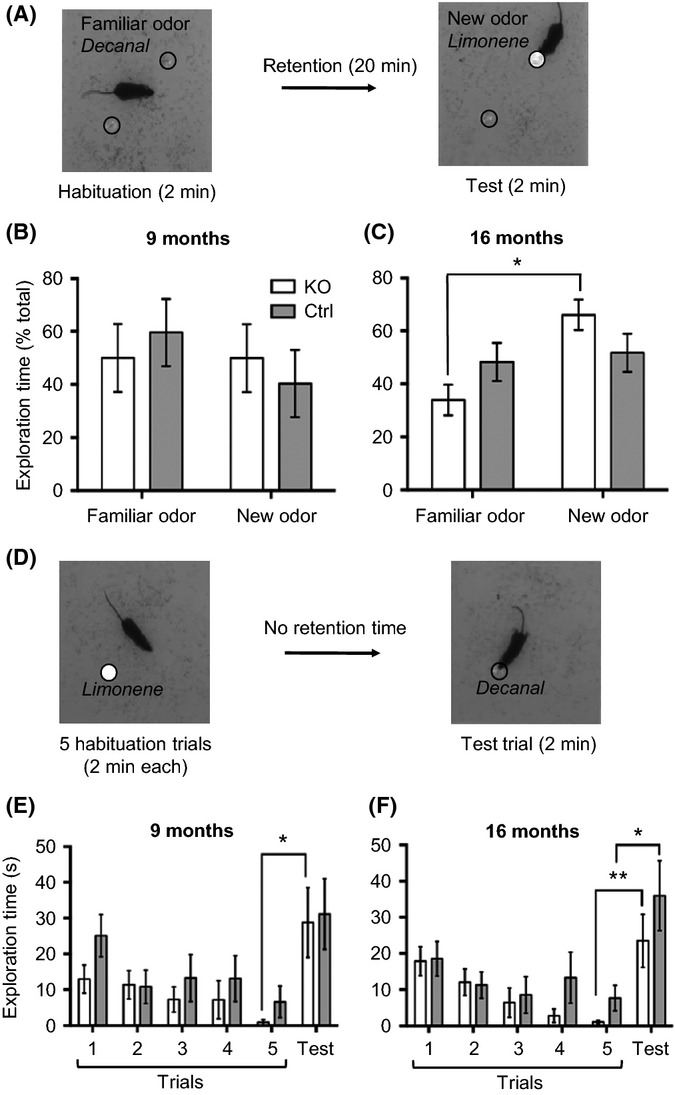
Aged mutants display improved olfactory function. (A–C) Olfactory memory was assessed in middle-aged (9 months, *n* = 14) and aged mutants (16 months, *n* = 28). (A) Design of olfactory memory test. Decanal was used as familiar odor and d-limonene as new odor introduced after 20-min retention time. (B and C) Preference index was calculated as time spent exploring the new odorant over total exploration time of both odors (as % of total). At 16 months, nonparametric paired Wilcoxon signed-rank test was performed to compare old and new odor preference index. *P*_*KO*_ = 0.013. (D–F) Olfactory learning and odor discrimination were assessed using habituation–dishabituation test. (D) Mice were exposed to d-limonene for 5 successive trials (learning phase) and to decanal in trial 6 (discrimination test). Statistical significance of discrimination was tested using Mann–Whitney *U*-test comparing exploration time in trials 5 and 6. (E) At 9 months, odor discrimination was significant for mutants (*P*_*Trial5–Trial6*_ = 0.018). A similar trend was observed for controls (*P*_*Trial5–Trial6*_ = 0.21). (F) Odor discrimination at 16 months (*P*_*Trial5–Trial6*_ = 0.007 for KO, *P*_*Trial5–Trial6*_ = 0.03 for controls). Mutants displayed significantly faster olfactory learning than controls. RM-ANOVA *P *=* *0.005 vs. *P *=* *0.55.

In the forebrain, adult-born neurons are involved in olfactory learning and memory (Sakamoto *et al*., 2011). To check whether increases in OB neurogenesis and glomerular complexity had an impact on olfactory function, we tested males at 9 and 16 months using an olfactory hole board (Mandairon *et al*., 2009). To assess olfactory memory, we used d-limonene and decanal. Animals were habituated to one odor and then exposed to the other after a long retention time of 20 min, so that aged mice were no longer able to differentiate new from familiar odors (Fig.5A). In these same experimental conditions, only young adult controls (3 months of age) succeeded in the recall test (new odor: 66% ±6 and old odor: 34% ±6 of total exploration time; *n* = 11, *P *=* *0.037, Wilcoxon signed-rank test). In contrast, and as expected, 9-month-old mutants and controls did not make any difference between the two odorants (Fig.5B). However, at 16 months, mutants preferentially recognized the new odor (Fig.5C), while controls still could not distinguish new from familiar. These results clearly demonstrated that the deletion of IGF-1R in adult NSCs rescued age-related impairment of short-term olfactory memory in male mutants. We next asked whether this gain-of-function phenotype resulted from more efficient learning or better odor discrimination. To answer that, we used a habituation–dishabituation test, exposing mice to d-limonene for five successive learning trials and then to decanal for the discrimination test (Fig.5D). As anticipated, all animals displayed efficient olfactory learning at 9 and 16 months (*P *=* *0.02; Fig. S5). Importantly, learning was significantly faster in mutants compared with controls at 16 months of age (Fig.5E,F). Mutants and controls displayed similarly efficient odor discrimination at 9 months (*P*_Trial5–Trial6_ = 0.018; Fig.5E) and at 16 months (*P*_*Trial5–Trial6*_ = 0.007 for KO and *P*_*Trial5–Trial6*_ = 0.03 for controls; Fig.5F). Thus, improved olfactory performance correlated with abundance of new neurons integrated into the olfactory bulb, and most likely resulted from faster olfactory learning. As these cognitive changes can also result from cell-autonomous structural modifications of IGF-1R-deficient neurons, we performed identical behavioral analyses in females, whose adult-born neurons were also IGF-1R knockout and displayed increased spine density, but without showing significant change in cell number. Importantly, female mutants did not show enhanced olfactory memory at 16 months (new odor, 52.8% ±9.3 of total exploration time; old odor, 47.2% ±9.4; Wilcoxon signed-rank test, *n* = 23, *NS*). This strongly suggested that improved olfactory function observed in aged male mutants was due to increased number of adult-born neurons and possibly to subsequent structural changes in olfactory bulb neurocircuitry, and not to differentiated IGF-1R^−/−^ neuronal phenotype.

### Suppression of IGF-1R in adult NSCs could prevent age-related metabolic changes

**Fig 6:**
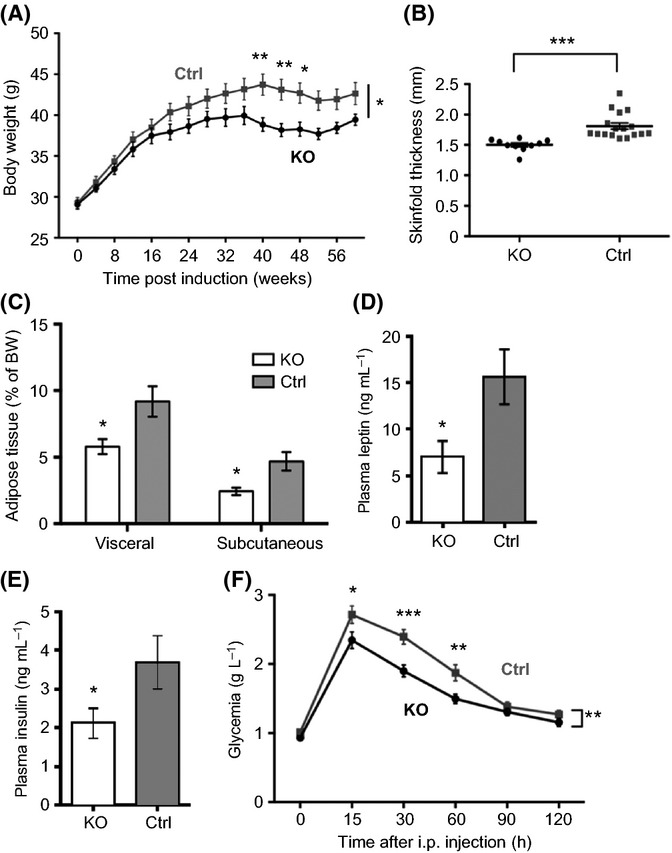
Metabolic shift in aged mutants. (A) Evolution of body weight during adult life. RM-ANOVA and Bonferroni *post hoc* test were used to compare KO and control data. *P *=* *0.039, *n* = 20. (B) Skin fold thickness. *P *<* *0.0001, Mann–Whitney *U*-test, *n*_*KO*_ = 10 and *n*_*Ctrl*_ = 17. (C) Adipose tissue (AT) mass expressed relative to body weight. *P *=* *0.026 for visceral AT, *P *=* *0.011 for subcutaneous AT, Mann–Whitney *U*-test, *n* = 7. (D and E) ELISA of plasma leptin (*P *=* *0.042, Mann–Whitney *U*-test, *n* = 10) and plasma insulin (*P *=* *0.032). (F) Glucose tolerance test was performed on animals fasted for 14 h. RM-ANOVA and Bonferroni *post hoc* test, *P *=* *0.004, *n* = 20. All analyzes were performed at 16 months.

Comparing data from male and female groups suggested functional relationships between number of adult-born new neurons in the OB, glomerular histoarchitecture, and olfactory performance. Consistent with these sex differences, mutant males also developed an intriguing metabolic phenotype at 16 months. Adult male mutants put on significantly less weight than controls (Fig.6A). At 9 months of age, when weight curves started to separate (Fig.6A), we detected slightly lower food intake in mutants (348 ± 15 vs. 399 ± 23 mg day^−1^ g^−1^ of BW; *n* = 7). However, this trend did not reach levels of significance and was no longer present at 16 months, leaving the persistent 12% body weight difference unexplained. We suspected that a change in metabolic homeostasis occurred in the aging mutant males. Indeed, at 16 months, mutant males had significantly thinner skin fold than controls (−17%, *P *<* *0.0001; Fig.6B) and dramatically decreased subcutaneous (−48%) and visceral (−37%) adipose tissue (AT) (*P *=* *0.011 and 0.026, respectively; Fig.6C). By subtracting AT mass from body weight, it became evident that AT was accounting for all the body weight difference between mutants and controls. In line with decreased lipid storage, circulating leptin was significantly low in mutants (−59%, *P *=* *0.042; Fig.6D). Moreover, mutants displayed decreased *ad libitum* plasma insulin (−42%, *P *=* *0.032; Fig.6E), and yet they managed tighter control of glycemia (Fig.6F), indicating enhanced insulin sensitivity. Mutants tended to be more active than controls, as revealed by actimetry and open field tests (Fig. S6); however, this did not suffice to explain the observed metabolic shift. The fact that only male mutants presented with metabolic changes suggested that rejuvenated olfactory neurogenesis and function entails relevant long-term alterations of glucose and lipid metabolism.

## Discussion

We demonstrated that IGF-I is a potent regulator of lifelong neuronal replacement in the mouse olfactory system. Blocking IGF-I signaling in adult NSCs delayed age-related depletion of stem cells and neuroblasts, resulting in enhanced neuronal production and integration. Two cellular mechanisms contributed to this pro-neurogenic effect. First, IGF-1R deletion slowed down proliferation in actively dividing progenitors, possibly increased stem cell self-renewal, and eventually postponed age-related attrition of the neuroblast compartment. Second, the inhibition of IGF-I signaling in adult-born neurons had a direct effect on neuronal differentiation and subsequent integration into olfactory bulb, potentially *via* downregulation of neuronal ERK signaling. In accord with that, a recent *in vivo* study showed that the deletion of Pten caused hyperactivation of the PI3K/Akt/mTOR pathway downstream of IGF-1R, leading to aberrant neuroblast differentiation and decreased integration in the OB (Zhu *et al*., 2012). Here, we produced evidence that IGF-1R-deficient neurons integrate more easily into preexisting olfactory circuitry, a finding potentially of high significance for cell grafting strategies.

Enhanced integration of IGF-1R knockout neurons was evident in both sexes, although effects were less significant in mutant females, probably because they started with less progenitors and neuroblasts at 4 months. This decreased number of neuronal precursors at short term may be due partly to the strong interaction between estrogen and IGF signaling pathways in females (Mendez *et al*., 2005). Deleting IGF-1R in adult NSCs would be sufficient to inhibit estrogen-mediated neuroprotection of neural progenitors, leading to acute depletion of this cell population (Luciani *et al*., 2012). Despite starting with fewer precursors than controls at 4 months, female mutants managed to accumulate more neurons in GL at 16 months. This discrepancy confirms that IGF-1R suppression in adult NSCs directly promotes survival and integration of adult-born neurons, independently from other effects on earlier stages of the lineage.

Interestingly, rejuvenating effects of IGF-1R mutation on neurogenesis developed only at long term. As energy budget constraints are critical to growth, maintenance, and aging in complex organisms (Kaplan & Robson, 2009), we described olfactory bulb neurogenesis mathematically, based on equations optimizing benefit/cost ratio. Predictions from simulations corroborated the late-life experimental phenotype, indicating that lowered *GF* becomes optimal specifically during the second half of life. Our findings contradict the long-standing belief that declining GH and IGF levels, characterizing individual aging in vertebrate species, result primarily from endocrine deficiency, a viewpoint frequently used to promote the conception of hormonal replacement (Sonntag *et al*., 2005). Much to the contrary, modeling results confirmed our experimental data, showing that diminished growth-factor-like signaling during adulthood can clearly be beneficial in long-term cell replacement strategies *in vivo*.

The idea of lowering growth-factor-like signals to enhance tissue homeostasis remains counterintuitive. In fact, lifelong tuning of stem cell self-renewal and differentiation is controlled by networks of growth signaling molecules such as PI3K, Akt, mTOR, and FoxO that are all important mediators of IGF-1R action (Groszer *et al*., 2001; Gan *et al*., 2008). Interestingly, FoxO transcription factors, which are activated when upstream IGF signals are blocked, preserve quiescence and prevent premature differentiation of neural progenitors (Renault *et al*., 2009). Accordingly, mice with multiple FoxO deficiency display initial postnatal increase in NSC proliferation, followed by a rapid decline of neurogenesis during adulthood (Paik *et al*., 2009). These reports support the idea that downregulation of Akt pathway as we observed in NSCs favors tissue maintenance. With our long-term study, we show for the first time *in vivo* that IGF-I signaling has differential and complementary effects on NSCs in SVZ, progenitors and neuroblasts in RMS, and neurons in OB. Our data strongly indicate that the suppression of IGF-1R in adult NSCs fosters neurogenesis by coordinating stem cell division pattern, neuroblast production, and neuronal integration over time.

Deletion of IGF-1R in adult NSCs enhanced neurogenesis in two cell layers of the adult bulb that present extremely different neuronal replacement rates. Indeed, in GCL, about 77% of neurons are replaced (0.80 × 10^6^ neurons of a total 1.04 × 10^6^), while in GL only 3% of neurons (0.05 × 10^6^ of 1.60 × 10^6^) are replaced (Parrish-Aungst *et al*., 2007). This means that IGF-I signaling is able to regulate lifelong neurogenesis over a wide range of cell replacement rates. Results obtained in adult mutant mice with low circulating GH and IGF-I, which display better maintenance of so-called very small embryonic-like cells in bone marrow (Ratajczak *et al*., 2011), corroborate the hypothesis that low IGF supports healthy tissue aging also in niches of peripheral tissues. In the same line, a mechanistic link between IGF signaling and HSC quiescence has been revealed in a recent study on *H19* imprinted gene (Venkatraman *et al*., 2013). Modification of *H19* maternal imprint specifically in HSCs activates IGF-I pathway, leading to FoxO3 phosphoactivation, to increased HSC proliferation at short term, and to their exhaustion at long term. Thus, continuously high IGF signals seem to be deleterious for blood tissue regeneration, a system with high cell turnover that differs in several aspects from the olfactory bulb neurogenic system.

Concomitant with the integration of many IGF-1R^−/−^ neurons into the OB, olfactory function improved and male mutants progressively developed a remarkable metabolic phenotype (Fig. S7). Aged mutants were leaner and exerted tighter control over glycemia, as indicated by low plasma leptin and insulin. Interestingly, the PI3K/mTOR pathway directly activates GLUT1 expression at the cell surface and intracellular glucose assimilation (Hennessy *et al*., 2005). Thus, high prevalence of IGF-1R^−/−^ neurons in the adult olfactory bulb mimics situations of food scarcity in an important sensory region of the mouse brain, and it is consistent that mutants show phenotypic changes typical for systemic adaption to restricted conditions. However, at this stage, our data do not suggest any particular mechanism explaining the link between OB neurogenesis and metabolic phenotype, as indicated by question marks in Fig. S7. In that supplementary figure, we present a synopsis of the present data and further interpretation. One interesting question is whether enhanced production of neurons in males is directly resulting from IGF-1R deletion in adult NSCs or is an indirect effect of modified food intake or activity. Although differences in diet and activity as we observed them appear minor compared with the drastic changes imposed by caloric restriction and animal training to produce changes in neurogenesis (Van Praag *et al*., 2005; Llorens-Martin *et al*., 2010; Park *et al*., 2013), we still checked this possibility and measured circulating levels of IGF-I, a major molecular effector of nutrition and exercise on adult neurogenesis. This revealed no difference between mutants and controls (KO: 560 ± 19 vs. controls: 541 ± 24 ng mL^−1^, *n* = 11–12), indicating that changes in neurogenesis were unlikely to be due to altered environment or inner milieu. Yet, it is possible that at least part of the metabolic phenotype observed in male mutants was of hypothalamic origin (Kokoeva *et al*., 2005; Lee *et al*., 2012). In that case, changes in hypothalamic neurogenesis could engender alterations in, for example, food intake or energy expenditure, and thereby modify body weight. Preliminary exploration of hypothalamic neurogenesis in our mutants (Z. Chaker and M. Holzenberger, unpublished data) shows that hypothalamic neurogenesis does continue until at least 9 months of age and that downregulation of IGF-I signaling in adult hypothalamic NSCs enhances production of new neurons also in this part of the brain. These data corroborate findings in the olfactory system.

Our mutants were clearly resistant to age-related obesity, notably exhibiting elevated insulin sensitivity, which reminds the metabolic features of some long-lived mutant mouse strains (Bartke & Westbrook, 2012). An evolutionary conserved relationship exists between chemosensory systems and aging, where insulin-like peptides (ILPs) are thought to be a central mechanistic link. Indeed, ILPs control olfactory learning in *Caenorhabditis elegans*, and specific gustatory neurons are known to regulate worm longevity through ILP signals (Alcedo & Kenyon, 2004; Chen *et al*., 2013). In *Drosophila*, olfactory food perception is sufficient to reverse lifespan extension of dietary-restricted flies (Libert *et al*., 2007). Thus, perception of environmental food resources modulates lifespan supposedly *via* insulin-like signals. In the same line, IGF-1R deletion in adult NSCs, which helps mutant animals adapt to conditions of restricted resources, might also change their aging trajectory.

Collectively, our findings unravel a lifelong physiological control of neuronal replacement by IGF signaling and clearly indicate that low IGF is beneficial for long-term maintenance of olfactory bulb structure and sensory function. This interventional study also suggests a potentially very interesting interdependence of sensory system and energy metabolism in mammals. Importantly, we show that downregulation of IGF-I signals constitutes a promising way to enhance neuronal production and integration during normal and possibly pathological aging of the mammalian brain.

## Experimental procedures

### Mouse models

Experimental protocols were approved by Comité d’éthique pour l’Expérimentation Animale ‘Charles Darwin’ (approval No. Ce5/2012/074). Control (nestin-CreER^T2^;CAG-TdTomato^+/0^;IGF-1R^wt/wt^) and mutant (nestin-CreER^T2^;CAG-TdTomato^+/0^;IGF-1R^flox/flox^) mice received tamoxifen (T5648, Sigma-Aldrich) at 12–13 weeks of age (84 mg kg^−1^ in 2 i.p. injections per day, for 5 consecutive days). Of critical note, the inducible Cre transgenic mouse used in this study has clearly different characteristics compared with the non-inducible nestin-Cre that we previously used for constitutive brain-specific knockout (Kappeler *et al*., 2008). Tamoxifen was dissolved in 10% ethanol in sunflower oil at 10 mg mL^−1^. Experimental groups of KO and control animals were analyzed 1, 6, and 13 months after induction of Cre recombination (4, 9, and 16 months of age, respectively), to discriminate between short- and long-term effects of the mutation. Efficiency of recombination was measured at 4 months of age, on migrating neuroblasts, using fluorescent tdTomato as reporter gene. Given the high efficiency of nestin-CreER^T2^, one month after Cre induction, all migrating neuroblasts were supposedly adult-born and tomato-positive. Recombination rate was determined from the ratio of tomato-positive neuroblasts (Tom^act^DCX^+^ fluorescent volume) vs. migrating neuroblasts (total DCX^+^ fluorescent volume).

### Immunohistochemistry

Mice were anesthetized and perfused transcardially with cold 4% PFA in 0.1 m PBS. Brains were postfixed overnight at 4 °C in 4% PFA and transferred to 30% sucrose in 0.1 m PBS for 36–48 h, until they sank. Samples were then snap-frozen in isopentane at −45 °C. Brains were sectioned into 30-μm slices on a freezing microtome in coronal (olfactory bulb) and sagittal plane (RMS). Cell-type-specific antibody staining was performed on free-floating sections and combined with Tom^act^ newborn cell lineage tracing. Mouse anti-rat nestin antibody (1:250, BD Pharmigen, Le Pont de Claix, France) was combined with rabbit polyclonal anti-glial fibrillary acidic protein antibody (GFAP, 1:2000, Dako Dakocytomation, Trappes, France) for stem cell identification. Other cell types were labeled with specific markers: mouse monoclonal anti-Mash1 antibody for neural progenitor identification (1:100, BD Pharmingen), antinuclear Ki67 antibody (1:250, Millipore, Molsheim, France) as proliferation marker, goat polyclonal anti-doublecortin antibody for neuroblast identification (DCX, 1:1000, Santa Cruz Biotechnology, Dallas TX, USA), and mouse monoclonal antineuronal marker antibody (NeuN 1:500, Millipore). For anti-Mash1 monoclonal antibody, blocking step used Dako’s Mouse On Mouse kit (BMK-2202). For all other antibodies, nonspecific binding was blocked with 10% serum (donkey or goat) and 0.3% Tween-20 in PBS for 45 min. Incubation with primary antibodies was performed with 3% serum and 0.3% Tween-20 overnight at 4 °C. Where possible, primary antibodies were simultaneously incubated (Mash1 and Ki67, for instance). Primary antibody incubation was followed by a 2-h incubation with fluorescent secondary antibodies (Alexa-488 or Alexa-633, Millipore). All sections were counterstained with a nuclear staining (DAPI or DRAQ5, 1:1000, Cell Signaling Technology, Danvers MA, USA) and the slides mounted in Mowiol (4-88 Mowiol powder, Sigma-Aldrich, Saint-Quentin-Fallavier, France). Microscopy and cell quantification were performed as detailed in Data S1 (Supporting information).

### Metabolic phenotyping

#### Biochemistry

Blood was collected from ocular sinus of conscious mice using EDTA, cooled on ice, centrifuged, and plasma-frozen. Plasma IGF-I, insulin, and leptin were measured by ELISA (R&D systems, Minneapolis MN, USA for IGF-I; Millipore for mouse leptin and insulin). Glucose tolerance test was performed on 14-h fasted animals. Mice were i.p. injected with 30% D-glucose (2 g kg^−1^ body weight), and glycemia was measured using OneTouch Ultra apparatus from tail blood 15, 30, 60, 90, and 120 min after injection.

#### Adipose tissue (AT) dissection

All AT was dissected from nonperfused animals and reported as subcutaneous (inguinal/dorsolumbar and interscapular) and visceral (gonadal, mesenteric and perirenal) compartments.

**Behavioral phenotyping** was performed as detailed in Data S1.

### Statistical analyses

All data are reported as mean ± SEM and statistical significance defined as *P *<* *0.05. Sample size *n* is the number of mice per experimental group. Mann–Whitney *U*-test was used for all nonparametric unpaired data sets. Student’s *t*-test was used for P-Akt IHC data and dendritic spine density measurements. ANOVA was used for repeated measures as indicated in figure legends. Wilcoxon signed-rank test was used for paired nonparametric data. *P <* *0.05, **P <* *0.01, ***P <* *0.001.

**Computational procedures** are detailed in supporting information.

## Supporting Information

**Fig. S1** Specificity of Tam-induced Cre-lox recombination. **Fig. S2** Deletion of IGF-1R in female NSCs leads to neural progenitor depletion at short-term while preserving neurogenic potential at long-term. **Fig. S3** Akt and ERK signaling pathways are differentially regulated in NSCs vs. differentiated neurons. **Fig. S4** Ubiquitous deletion of IGF-1R in the adult brain induces dramatic remodeling of olfactory glomeruli. **Fig. S5** Faster olfactory learning in mutant animals. **Fig. S6** Aged mutants display no difference in overall activity. **Fig. S7** Synopsis of results.

**Data S1** Experimental procedures.
