# Supplementary material for: Suppression of IGF-I signals in neural stem cells enhances neurogenesis and olfactory function during aging
Source: Aging Cell. 2015 Jul 29;14(5):847–56. doi: 10.1111/acel.12365 (PMC4568972; doi:10.1111/acel.12365)
Supplement: Supplementary file 1 [file acel0014-0847-sd1.pdf]

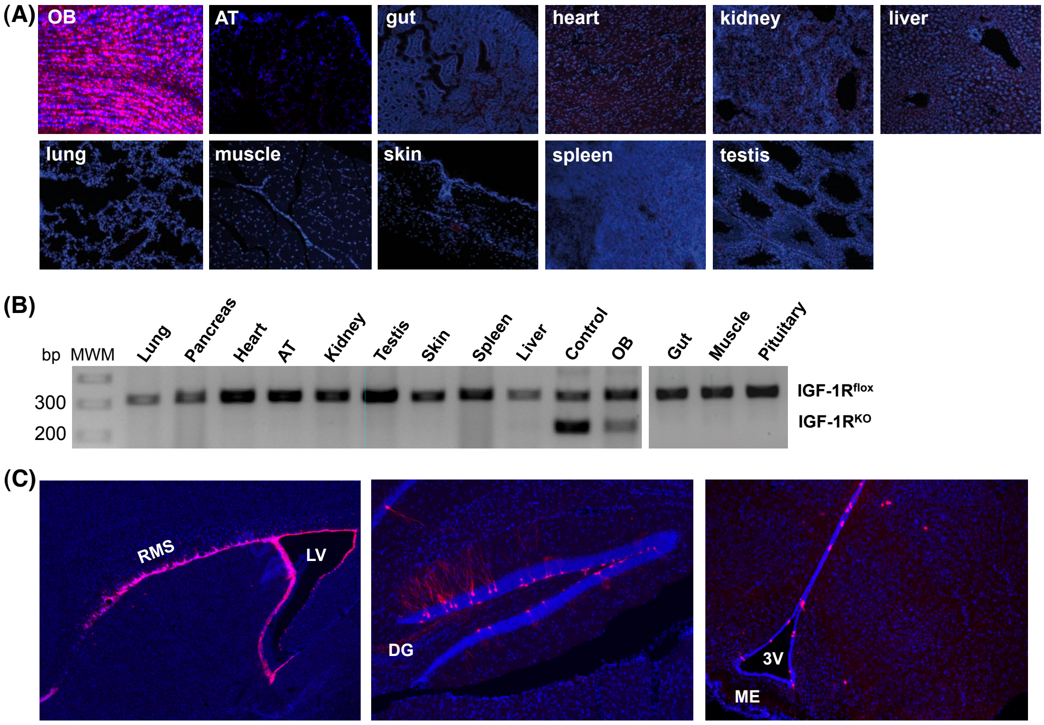
(A)
OB
AT
gut
heart
kidney
liver
lung
muscle
skin
spleen
testis
(B)
bp
MWM
Lung
Pancreas
Heart
AT
Kidney
Testis
Skin
Spleen
Liver
Control
OB
Gut
Muscle
Pituitary
300
200
IGF-1R flox
IGF-1R KO
(C)
RMS
LV
DG
3V
ME

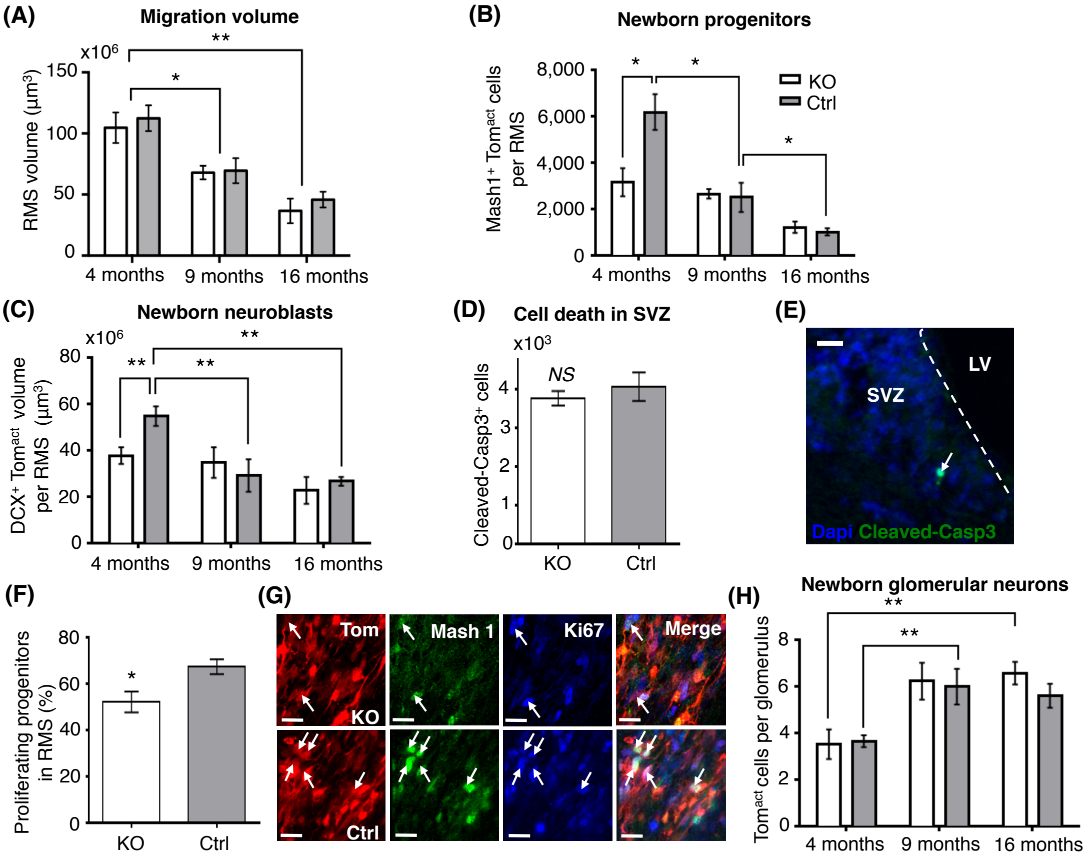

Figure S2

Chaker *et al.*

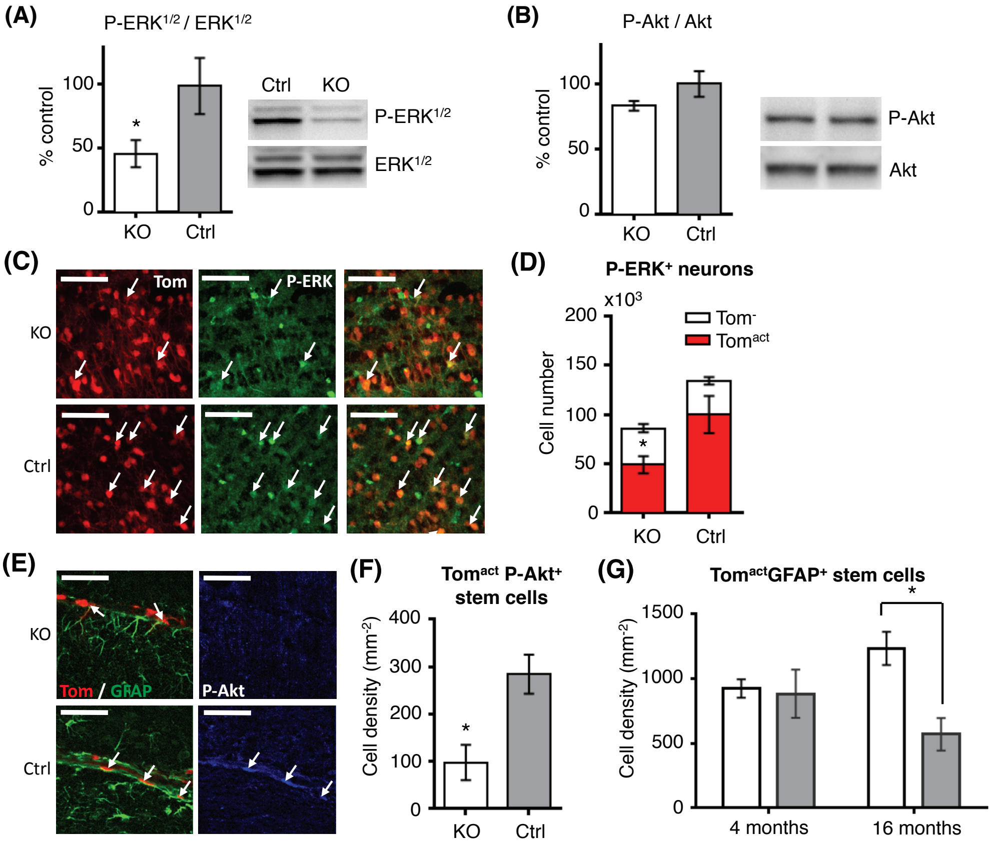

(A)
P-ERK1/2 / ERK1/2
% control
100
50
0
*
KO
Ctrl
Ctrl
KO
P-ERK1/2
ERK1/2
(B)
P-Akt / Akt
% control
100
50
0
KO
Ctrl
P-Akt
Akt
(C)
Tom
P-ERK
KO
Ctrl
(D)
P-ERK+ neurons
x10³
Cell number
200
150
100
50
0
Tom-
Tomact
*
KO
Ctrl
(E)
KO
Ctrl
Tom / GFAP
P-Akt
(F)
Tomact P-Akt+ stem cells
Cell density (mm-2)
400
300
200
100
0
*
KO
Ctrl
(G)
TomactGFAP+ stem cells
Cell density (mm-2)
1500
1000
500
0
*
4 months
16 months

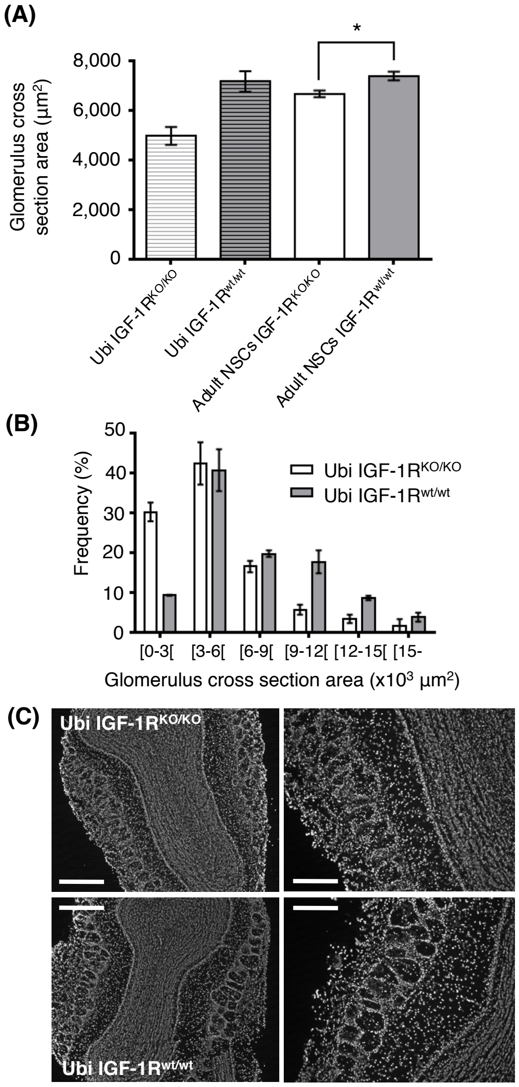
(A)
*
8,000
6,000
4,000
2,000
0
Glomerulus cross section area (µm2)
Ubi IGF-1RKO/KO
Ubi IGF-1Rwt/wt
Adult NSCs IGF-1RKO/KO
Adult NSCs IGF-1Rwt/wt
(B)
50
40
30
20
10
0
Frequency (%)
Ubi IGF-1RKO/KO
Ubi IGF-1Rwt/wt
[0-3[
[3-6[
[6-9[
[9-12[
[12-15[
[15-
Glomerulus cross section area (x103 µm2)
(C)
Ubi IGF-1RKO/KO
Ubi IGF-1Rwt/wt

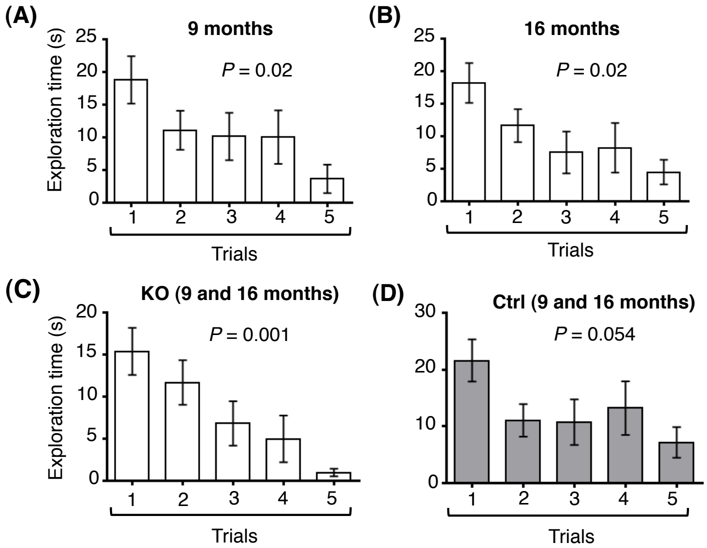
(A)
9 months
Exploration time (s)
P = 0.02
Trials
(B)
16 months
P = 0.02
Trials
(C)
KO (9 and 16 months)
Exploration time (s)
P = 0.001
Trials
(D)
Ctrl (9 and 16 months)
P = 0.054
Trials

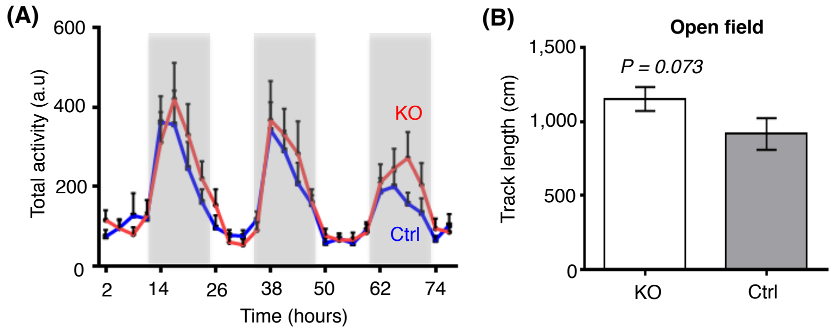

Figure S6

Chaker *et al.*

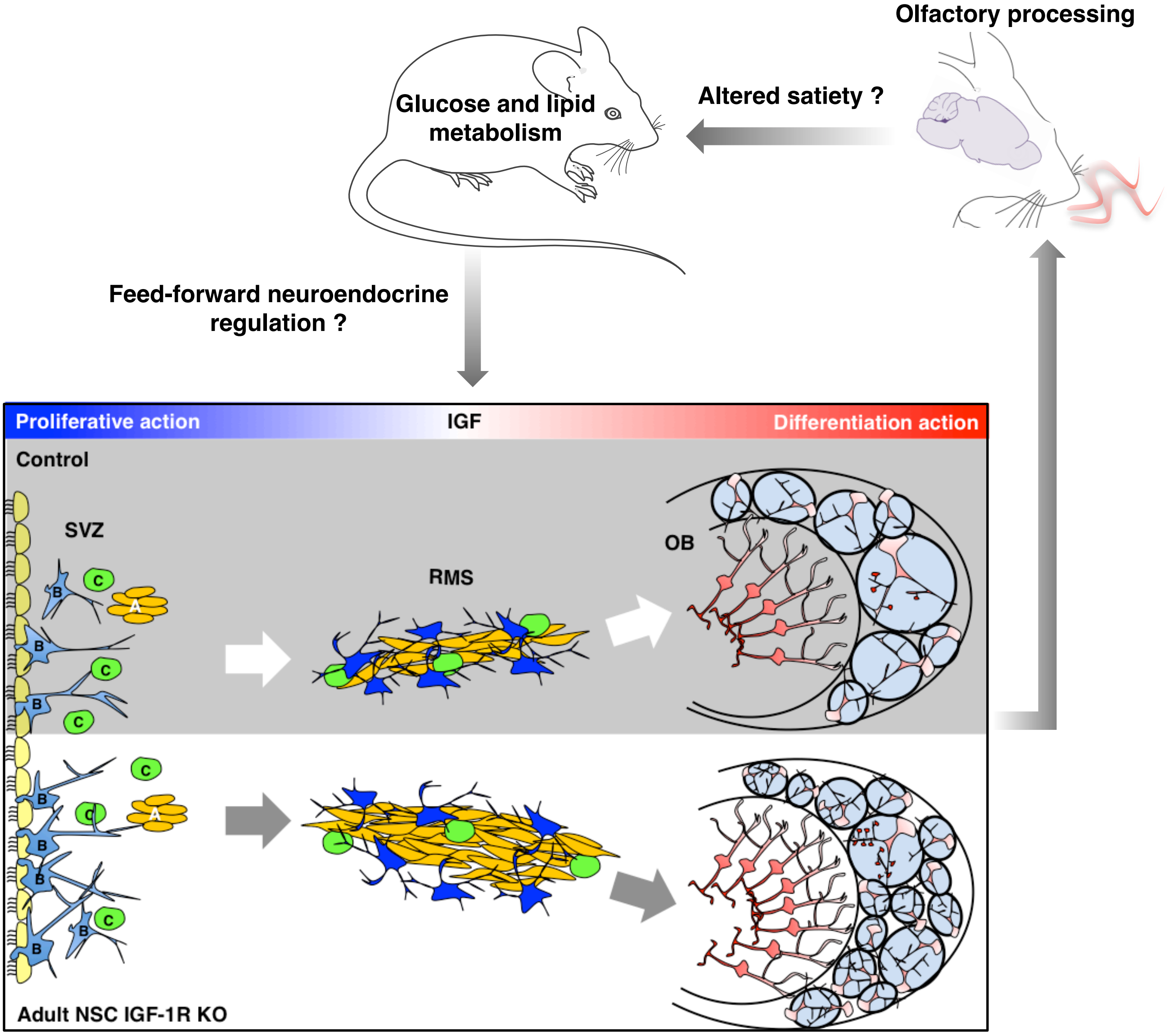

Figure S7

Chaker *et al.*
