# Supplementary material for: Suppression of IGF-I signals in neural stem cells enhances neurogenesis and olfactory function during aging
Source: Aging Cell. 2015 Jul 29;14(5):847–56. doi: 10.1111/acel.12365 (PMC4568972; doi:10.1111/acel.12365)
Supplement: Supplementary file 2 [file acel0014-0847-sd2.docx]

SUPPORTING INFORMATION

**Supporting figure legends**

**Fig. S1. Specificity of Tam-induced Cre-lox recombination.** (A) Representative micrographs of peripheral tissues from a 23-month-old nestin-CreER^T2^;CAG-TdTomato^+/0^;IGF-1R^KO/KO^ mutant mouse, Tam-induced at 3 months of age. Tissue sections were counterstained with DAPI. Strong tdTomato expression was primarily restricted to OB. Cautious scrutiny of numerous tissue sections confirmed that on average < 0.1% of cells were recombined in peripheral tissues. Scale bars, 100 μm. (B) Genomic PCR on a panel of tissue samples confirmed absence of Cre-lox gene excision in peripheral tissues. A control sample from heterozygous IGF-1R^flox/KO^ animal (50% of alleles recombined) served as positive control. (C) tdTomato expression in the 3 neurogenic niches of the adult brain, namely SVZ and RMS (left panel; LV, lateral ventricle), dentate gyrus (center panel; DG), and hypothalamus (right panel; 3V, third ventricle; ME, median eminence). Scale bars, 200 μm.

**Fig. S2. Deletion of IGF-1R in female NSCs leads to neural progenitor depletion at short-term while preserving neurogenic potential at long-term.** (A) RMS volume was measured on DAPI counterstained sections. *P_4Mo-16Mo_ =* 0.006 for KO and *P_4Mo-16Mo_* = 0.001 for control, M-W *U*-test. n = 6 at 4 months, n = 3 at 9 months, n_KO_ = 3 and n_Ctrl_ = 6 at 16 months. (B and C) At 4 months, the number of Tom^act^ progenitors and Tom^act^ neuroblasts was lower in mutant females than in controls. *P* = 0.026 (B) and *P* = 0.009 (C). At long-term, only control females displayed continuous depletion of progenitors and neuroblasts. Progenitors (B): *P_4Mo-9Mo_* = 0.048 and *P_9Mo-16Mo_* = 0.024; neuroblasts (C): *P_4Mo-9Mo_* = 0.009; M-W *U*-test, n = 6. (D) Prevalence of cell death following IGF-1R deletion in adult NSCs was determined by counting cleaved-casp3^+^ cells in the SVZ. *P* = 0.73, M-W *U*-test, n = 7. (E) Arrow indicates a cleaved-casp3^+^ cell on DAPI counterstained SVZ coronal section. Scale bar, 20 μm. (F) At 4 months of age, the proportion of proliferating newborn neural progenitors in the RMS was measured as the ratio of triple-labeled Tom^act^Mash1^+^Ki67^+^ cells over total Tom^act^Mash1^+^ progenitors. *P* = 0.014, M-W *U*-test, n = 6. (G) Representative micrographs of sagittal RMS sections. Arrows indicate triple-labeled progenitors. Scale bars, 20 μm. (H) Newborn glomerular cells per glomerulus accumulated with age. *P_4Mo-16Mo_* = 0.036 for KO and *P_4Mo-16Mo_* = 0.002 for controls, M-W *U*-test.

**Fig. S3. Akt and ERK signaling pathways are differentially regulated in NSCs *versus* differentiated neurons.** (A and B) Western blot analyses of OB homogenates from 16-month-old males. (A) P-ERK^1/2^/ERK^1/2^-ratio was normalized to control. *P* < 0.05, M-W U-test, n = 5-6. (B) P-Akt/Akt ratio normalized to control. *NS*. (C) Representative micrographs of GCL sections at 16 months. Arrows indicate Tom^act^P-ERK^+^ cells. Scale bars, 50 μm. (D) Quantification of P-ERK^+^ cell population in OB at 16 months, composed of Tom^act^ (red bars) and Tom^-^ (white) neurons. Tom^act^P-ERK^+^ cell number was significantly decreased in mutants. *P* < 0.05, M-W *U*-test, n = 6). (E) Representative micrographs of SVZ sections at 4 months. Arrows indicate Tom^act^GFAP^+^ stem cells on left panels and P-Akt^+^ stem cells on the right. Scale bars, 50 μm. (F) Quantification of Tom^act^GFAP^+^P-Akt^+^ cell density around the SVZ at 4 months. *P* < 0.05, Student’s *t*-test, n = 3. (G) Comparison of Tom^act^GFAP^+^ stem cell density in SVZ at 4 and 16 months. *P* < 0.05, M-W U-test, n = 5.

**Fig. S4. Ubiquitous deletion of IGF-1R in the adult brain induces dramatic remodeling of olfactory glomeruli.** (A) Glomerulus size in mice with ubiquitous adult IGF-1R knockout. Ubi-IGF-1R^KO/KO^: 4,971 ± 359 μm^2^, n = 2; and adult NSC specific KO: 6,664 ± 138 μm^2^, n = 7. (B) Glomerulus size distribution in Ubi-IGF-1R^KO/KO^ and Ubi-IGF-1R^wt/wt^. n = 2 with >80 glomeruli measured per animal. (C) Left micrographs (scale bars, 500 μm): representative DAPI-counterstained coronal sections of Ubi-IGF-1R^KO/KO^ and Ubi-IGF-1R^wt/wt^ olfactory bulbs. Right (scale bars, 250 μm): higher magnification of GL.

**Fig. S5. Faster olfactory learning in mutant animals.** (A and B) Olfactory learning data pooled for KO and control animals at 9 (A) and at 16 months (C). R-M ANOVA was significant for both ages, *P* = 0.02. (C and D) Olfactory learning data pooled for age, comparing KO (C) with control (D) animals. R-M ANOVA revealed that mutants learned significantly faster, *P_KO_* = 0.001 and *P_Ctrl_* = 0.054.

**Fig. S6. Aged mutants display no difference in overall activity.** (A) Activity was recorded individually for 84 h under 12-h light/12-h dark cycle. n = 12, dark phases shaded. Curves show sliding means for 3-h intervals. (B) Track length in open field test reflects exploration propensity. *P* = 0.073, M-W *U*-test, n = 20.

**Fig. S7. Synopsis of results.** Deletion of IGF-1R in adult NSCs delayed age-related depletion of neuroblasts (type A cell) in the RMS with no effect on the number of migrating neural progenitors (type C), and increased stem cell maintenance (type B) in the niche. As a consequence, more newborn neurons integrated into GCL and GL, and mutants displayed significantly more and smaller glomeruli. In mutants, adult-born glomerular neurons formed more synapses in pre-existing olfactory networks than controls. These cellular and histoanatomical changes improved olfactory function in mutant mice, possibly modifying satiety, and metabolic homeostasis (arrows with question mark). Mutants were significantly leaner and more insulin-sensitive. Together, these elements may function as an auto-adaptive feed-forward regulation depending on local IGF secretion in SVZ, RMS and OB.

**Supporting experimental procedures**

***Mouse genetics and husbandry.*** All analyses have been performed on males and females. We backcrossed CAG-tdTomato^+/0^ mice (*http://jaxmice.jax.org/strain/007908.html*) to 129/SvPas (129) genetic background. Founder group A was produced crossing CAG-tdTomato^+/0^ with knock-in IGF-1R^flox/flox^ mice on 129 background. Founder group B was obtained crossing nestin-CreER^T2^ transgenic mice with C57BL/6 (B6) background (provided by Dr. Amelia J. Eisch) with IGF-1R^flox/flox^ (B6) mice. The final triple transgenic mutants (nestin-CreER^T2^;CAG-tdTomato^+/0^;IGF-1R^flox/flox^) were generated by crossing founders A and B, resulting in fully reproducible F1 hybrid B6/129 background. In parallel, age-matched control animals were produced on the same F1 hybrid background by crossing nestin-CreER^T2^ (B6) with CAG-TdTomato^+/0^ (129) mice (nestin-CreER^T2^;CAG-TdTomato^+/0^;IGF-1R^wt/wt^). All mice were housed in IVCs under SPF conditions at 21-23 °C, 12 h/12 h light/dark cycle, and food and water *ad libitum*.

***Microscopy and cell quantification.*** Fluorescent images were collected using a spinning disk microscope (Yokogawa spinning head, Roper/Leica), equipped with a QuantEMCCD 512SC camera from Photometrics and Metamorph 7.5 for image acquisition. An unbiased quantification protocol, inspired from optical fractionator and stereological methods (Coggeshall 1992), allowed precise measurement of absolute number of cells for each region of interest. For all RMS quantifications, brains were sectioned sagitally from 0.4 mm medial to 1.44 mm lateral. This sampling region includes the entire RMS. Sections were collected in 8 parallel sets of four, with sections being 240 µm apart. All 8 sets represented comparable samples of the entire region, and each cell-type-specific staining was performed on a given set. Sampling was fully reproducible between animals. Cell densities were estimated on Z-stacks composed of 2-4 µm optical slices. Using the method of Cavalieri, the total volume of interest was determined and the absolute number of cells per region calculated. The number of Tom^act^ cells per glomerulus was quantified from sagittal sections of olfactory bulbs, using an epifluorescence microscope (DM5000B, Leica). We sampled a minimum of 50 glomeruli per animal, on 4 sagittal sections. Cells were manually counted using Z-stack treatment options of FIJI software (*http://fiji.sc/Fiji)*. For automated quantification, we used the colocalization plugin to compare Tom^act^ versus DCX^+^ fluorescence volumes.

***Olfactory bulb (OB) histoanatomy.*** OBs from 16-month-old animals were sectioned coronally, starting at 1.35 mm from the OB edge. Six sets of 30-µm-sections being 180 µm apart, were collected. Total OB glomerular and granular volumes were determined using epifluorescence micrographs. Glomerulus size was averaged from approximately 180 medio-lateral glomeruli per animal, and data used to establish size frequency distribution. Newborn cells (Tom^act^) in granular cell layer were counted on 6 coronal OB sections using spinning disk Z-stacks.

***Dendritic morphology.*** An average number of 4 secondary dendrites was analyzed per animal by acquisition of Z-stacks of 25-50 optical sections at 0.5 µm intervals, with a 100x spinning disk microscopy lens. Spine density was determined by manual analysis of individual Z-slices and 3D reconstructions of intact dendritic arbors.

***Western immunoblot.*** Snap-frozen GCL samples were homogenized in buffer (25 mM tris–HCl, 150 mM NaCl, 1% Triton X-100, 0.5% sodium deoxycholate, 0.1% SDS, 1 mM EDTA, 1 mM EGTA) with protease and phosphatase inhibitors (Roche). 30 μg protein were loaded onto tris-glycine 4-20% gradient gels (Bio-Rad), migrated at 100 V, and electro-transferred to PVDF membrane. We used antibodies against Akt and phospho-Akt Ser473 (1:1000, CST), p44/42 MAPK ERK (1/2) and phospho-ERK Thr202/Tyr204 (1:1000, CST).

***Behavioral phenotyping.*** We used automated 3-point video tracking (Viewer, Biobserve).

***Olfactory memory*.** The hole-board olfactory test consisted of a flat square board (40 x 40 cm white PMMA) with two holes of 30 mm diameter (Mandairon et al. 2009). A polypropylene swab impregnated (or not) with odorant was placed in a glass beaker fixed below each hole, a stainless steel grid preventing contact. Odorants used were d-limonene (pure) and decanal (diluted in mineral oil to 1.78%, Sigma Aldrich). Prior to each test, mice were acclimated to the behavioral room in individual cages for at least 15 min. The short-term memory test consisted of three trials: habituation, acquisition and recall phase. No distant visual cues allowing spatial orientation were present in the experimental room. The mouse was placed on the board and allowed to explore for 2 min, with no odor in holes. The animal was returned to its home cage for 1 min, and then placed again on the board for 2 min with decanal in both holes. It was then placed in its home cage for 20 min (retention time), and tested again on the board for 2 min with one hole containing a swab impregnated with decanal (familiar), the other with +limonene (new odor). The mouse was considered exploring the odor if (1) its nose was less than 1 cm from the edge of the hole, and (2) the angle between midline of the head and line between center of the hole and nose was superior to 90. We observed no difference in overall exploration between groups, which is expected and a prerequisite for validity of the test. The pair of odorants used had been chosen after careful screening of several odors. This screening aimed at (1) optimizing discrimination, by trying out different concentrations to maximize detection threshold (Mandairon et al. 2009), and (2) excluding that in the final pair of odors one of them was preferred. For all experiments, new and familiar odors were randomized.

***Habituation-dishabituation test*.** This paradigm assesses (1) the ability of animals to habituate to a given odor (d-limonene), and (2) their capacity to discriminate between this and a novel odor (decanal). An olfactory board with a single hole was used. Before each test, mice were acclimated to the room in individual cages. No distant visual cues were available. Each mouse was exposed to d-limonene for 5 consecutive 2-minute trials, separated by 5-minute intervals in the home cage. The odor swab was changed for the last trial, and the mouse exposed to the new odorant decanal. Exploration time, expected to decrease during the habituation trials and to increase during discrimination test, was determined. Exploration criteria were the same as for olfactory memory test.

***Open field*.** Animals were gently placed in the open field (50 x 35 x 35 cm opaque white arena) and left to explore for 10 min. Total distance travelled, and time spent in wall or center zones were analyzed.

**Supporting computational procedures**

***Model framework (Matlab software)*.** We built a dynamical system of four populations: stem cells (S), progenitors (P), neuroblasts (B) and neurons (N). A fifth compartment of astrocytes (A) served as support cells for P, B and N populations. We modeled self-renewal and differentiation dynamics using multi-compartment ordinary differential equations (ODEs). Death, renewal and differentiation rates depend on time (aging) and growth-factor-like stimulation (*GF*).

***Neurogenesis dynamics*.** Each population *x* is characterized by self-renewal $\mu_{x}^{SR}$, differentiation $\mu_{x}^{D}$ and death rates $d_{x}$, that together define population growth rate $\gamma_{x}$, according to:

$\gamma_{x}\left( t,GF \right)=\mu_{x}^{SR}\left( t,GF \right)-{\beta_{x}\mu}_{x}^{D}\left( t,GF \right)-d_{x}(t,GF)$ , $\forall x\in\left\{ S,P,B \right\}$ (1)

where $\beta_{x}$ is a stochiometric factor accounting for possible asymmetrical stem cell differentiation. The model thus writes (dropping dependences on time and *GF* for readability):

$$\left\{ {\frac{dS}{dt}=\gamma_{S}S\left( 1-\sigma\left( \gamma_{S} \right)\frac{S}{K_{S}\left( S,S \right)} \right) \atop\begin{aligned} \frac{dP}{dt}=\left( 2-\alpha\right)\mu_{S}^{D}S+\gamma_{P}P\left( 1-\text{σ}\left( \gamma_{P} \right)\frac{P}{K_{P}\left( A,P \right)} \right) \\ \frac{dB}{dt}=2\mu_{P}^{D}P+\gamma_{B}B\left( 1-\text{σ}\left( \gamma_{B} \right)\frac{B}{K_{B}\left( A,B \right)} \right) \\ \frac{dN}{dt}=\mu_{B}^{D}B-d_{N}N\left( 1+\frac{N}{K_{N}\left( A,N \right)} \right) \\ \frac{dA}{dt}=\frac{S}{\mu_{S}^{SR}+\mu_{S}^{D}} \end{aligned}} \right.$$

where $\sigma\left( x \right)$ is the signum function ($\sigma\left( x \right)=1$ if $x\geq0$, -1 else) and $K_{x}( )$ constants are carrying capacities (defined below). In the olfactory system, adult stem cells differentiate mostly asymmetrically ($S\to S+P$), thus α is set at 0.9.

***Division, differentiation and death rates*.** Dependency on *GF* is cell-type-specific, but the effect of aging is assumed to be the same for all cell types. For *S* and *P* cells, self-renewal rates decrease with age and increase when *GF* is low, concordantly with FOXO regulation of adult NSCs (Renault et al. 2010).

$\mu_{x}^{SR}\left( t,GF \right)=a\exp\left( c{GF}^{2}-bt^{2} \right), \forall x\in\left\{ S,P \right\}$ (3)

where *a*, *b*, and *c* are constants (see table below).

The equation describing *GF* regulation of neuroblast self-renewal is different. The proliferative effect of *GF* is stronger on primary regenerating compartments than on more differentiated ones, which we translate as:

$\mu_{B}^{SR}\left( t,GF \right)=a\exp\left( -bt^{2} \right){GF}^{2}$ (4)

Differentiation rates increase when *GF* is high, and decrease with age.

$\mu_{x}^{D}\left( t,GF \right)=d\exp\left( -jt^{2} \right){GF}^{2}, \forall x\in\left\{ S,P,B \right\}$ (5)

Differentiation and self-renewal rates decrease by square time and increase by square *GF*, because rate derivative (pace of increase) should also be depending on time and *GF*.

Death rate decreases when *GF* is high (pro-survival factor) and increases with age.

$d_{x}\left( t,GF \right)=n\exp\left( -\left( mt+zGF \right) \right), \forall x\in\left\{ S,P,B,N \right\}$ (6)

|  | ***S*** | ***P*** | ***B*** | ***N*** |
| --- | --- | --- | --- | --- |
| **a** | 0.028 | 0.4 | 0.15 | - |
| **b** | 2.5 × 10^-6^ | 5.0 × 10^-6^ | 5.0 × 10^-6^ | - |
| **c** | -1 | -1 | - | - |
| **d** | 0.072 | 0.4 | 0.15 | - |
| **j** | 2.5 × 10^-6^ | 5.0 × 10^-6^ | 5.0 × 10^-6^ | - |
| **n** | 0.05 | 0.05 | 0.05 | 0.01 |
| **m** | -1.5 × 10^-3^ | -1.5 × 10^-3^ | -1.5 × 10^-3^ | -5.0 × 10^-6^ |
| **z** | 3 | 3 | 3 | 3 |

These parameters were inspired from polynomial fits of hippocampal stem/progenitor content and decay rates developed by Encinas *et al* (Encinas et al. 2011).

***Age-related astrogliosis*.** Astrocyte dynamics follow the astrocytic division coupled hypothesis developed for hippocampal NSCs (Encinas et al. 2011). Age-related increase of astrocyte number results from successive SC divisions.

***Carrying capacity and cellular cost definition***. Experimental evidence for cell-specific energetic cost being difficult to obtain, we assumed that the cost per unit of time was increased when cell turnover was high. For *P*, *B* and *N* populations, carrying capacities are expressed as follows: $K_{x}=AR/Cost$, where *R* represents available resources (a value comprised between 0 and 1) and *A* is the astrocyte population. We set it at the maximum (*R* = 1). A more specific microenvironment is supporting stem cell fate choice. This small niche (where stem cell-to-cell interactions play an important role) was modeled differently: $K_{S}=SR/Cost$, which can be read as $K_{S}=R'/Cost$ (with *R*’<<*R*). Cell turnover r and population cost are expressed as follows:

$\left\{ \begin{aligned} r\left( t,GF \right)=\mu_{x}^{SR}\left( t,GF \right)+\mu_{x}^{D}\left( t,GF \right)+d_{x}(t,GF) \\ Cost\left( x,t,GF \right)=\frac{1}{1+\text{exp}\left( \frac{5-\sqrt{x(t,GF)}}{10r(t,GF)} \right)} \end{aligned} \right.$ (7)

***Initial conditions*.** The model is describing adult neurogenesis and starts with proportionate cell ratios according to our experimental data and to the literature. Number of migrating progenitors is 6 × 10^3^ cells. Supposing that average neuroblast cell volume is about 80-100 μm^3^ (about 3 times smaller than a neuron), we estimated total number of migrating neuroblasts to 500-600 thousand cells (B_0_ = 100*P_0_). Total number of olfactory bulb neurons is around 3.5 million cells (Parrish-Aungst et al. 2007), we then set N_0_ = 10*B_0_. In the adult rodent brain, glia:neuron ratio ranges from 0.5:1 to 2:1 (Parrish-Aungst et al. 2007; Bandeira et al. 2009), we set A_0_ = 2*N_0_. Exact number of stem cells was not measured experimentally. We assume it to be in the same range as neural progenitor number (S_0_ = P_0_).

$$\left\{ \begin{aligned} \begin{aligned} \begin{aligned} \begin{aligned} S\left( 0 \right)=1 \\ P\left( 0 \right)=1 \end{aligned} \\ B\left( 0 \right)=100 \end{aligned} \\ N\left( 0 \right)=1000 \end{aligned} \\ A\left( 0 \right)=2000 \end{aligned} \right.$$

***Neuronal function*.** At each time step D*t*, the model selects the amount of *GF* needed to maximize a benefit/cost ratio. To compute benefit at each time step, we first round the *N* variable to closest integer to obtain the corresponding number of neurons. The benefit provided by each individual neuron is then attributed at random according to a normal distribution *FI* whose mean (*M*) and standard deviation (*Var*) depended on time (see equation 4). *M* was decreasing with time, mimicking age-related decline of neuronal functionality. Neuronal heterogeneity *Var* was increasing with age.

$$\left\{ \begin{aligned} M\left( t \right)=1-\frac{t}{t+1000} \\ \text{Var}\left( t \right)=0.1\left( 1+\frac{t}{t+1000} \right) \end{aligned} \right.$$

Total benefit is calculated as the sum of individual functional indexes FI.

***Optimization procedure*.** The optimization procedure was sequential. At each integration step “Dt”, the model optimized benefit over cost ratio (B/C) by screening all possible *GF* values and then resulting population sizes (*S, P, B, N*) at time t+Dt. Once the optimal *GF* value found, each population size was modified accordingly and the model moved on to step t+Dt. *GF* final distribution was smoothed using a sliding window of 36 Dt-width.

***Model without optimization*.** A second model using the same parameters but running with a fixed *GF* value throughout lifespan served as negative control for the optimization procedure. By confronting both models, we observed that B/C reached after optimization was similar to the *GF* = 1 situation during the first 2 thirds of life (Fig. CPa). During this same period, optimized B/C was higher than the *GF* = 0.8 or *GF* = 0.1 situation. Interestingly, our optimization procedure led to higher B/C ratio compared to abundant constant *GF*, at the last third of life specifically (Fig. CPb). At this late-life stage, cost was minimal with optimization compared to very low (0.1), intermediate (0.8) or maximum (1) *GF* values (Figure CP3.2b).


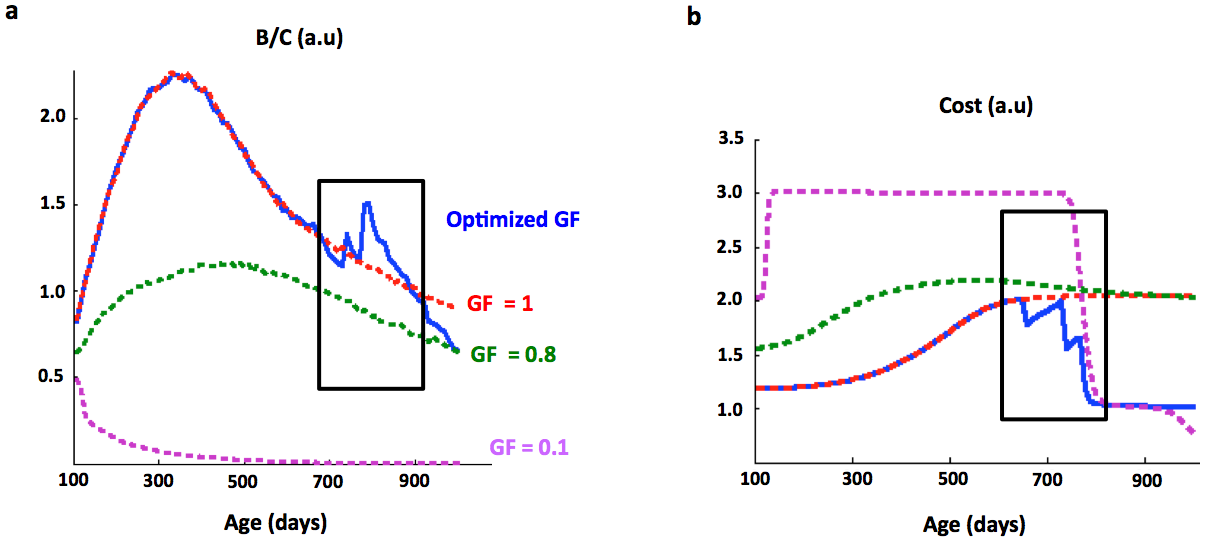


**Fig. CP**. Benefit/Cost ratio (B/C) as fitness parameter in optimized *vs* unconstrained model. Blue curves represent simulations with optimization procedure. Red, green and purple curves are results of the unconstrained model with *GF* = 1, *GF* = 0.8 and *GF* = 0.1 respectively. **a,** B/C evolution over age. The black square highlights the moment when B/C becomes highest in the optimized model. **b,** Optimized model minimizes cost at the last third of life (black square).

**Supporting references**

Bandeira F, Lent R & Herculano-Houzel S (2009) Changing numbers of neuronal and non-neuronal cells underlie postnatal brain growth in the rat. *Proc. Natl. Acad. Sci. U. S. A.* 106, 14108–14113.

Coggeshall RE (1992) A consideration of neural counting methods. *Trends Neurosci.* 15, 9–13.

Encinas JM, Michurina T V, Peunova N, Park J-H, Tordo J, Peterson DA, Fishell G, Koulakov A & Enikolopov G (2011) Division-coupled astrocytic differentiation and age-related depletion of neural stem cells in the adult hippocampus. *Cell Stem Cell* 8, 566–579.

Mandairon N, Sultan S, Rey N, Kermen F, Moreno M, Busto G, Farget V, Messaoudi B, Thevenet M & Didier A (2009) A computer-assisted odorized hole-board for testing olfactory perception in mice. *J. Neurosci. Methods* 180, 296–303.

Parrish-Aungst S, Shipley MT, Erdelyi F, Szabo G & Puche AC (2007) Quantitative analysis of neuronal diversity in the mouse olfactory bulb. *J. Comp. Neurol.* 501, 825–836.

Renault VM, Rafalski VA, Morgan AA, Salih DAM, Jamie O, Webb AE, Villeda SA, Thekkat PU, Guillerey C, Denko NC, Palmer TD, Butte AJ, Brunet A, Drive P & Ca S (2009) FoxO3 regulates neural stem cell homeostasis. *Cell Stem Cell*. 5, 527–539.
